# Analyses of the Cellular Interactions between the Ossification of Collagen-Based Barrier Membranes and the Underlying Bone Defects

**DOI:** 10.3390/ijms24076833

**Published:** 2023-04-06

**Authors:** Said Alkildani, Yanru Ren, Luo Liu, Denis Rimashevskiy, Reinhard Schnettler, Milena Radenković, Stevo Najman, Sanja Stojanović, Ole Jung, Mike Barbeck

**Affiliations:** 1 BerlinAnalytix GmbH, 12109 Berlin, Germany; 2 Clinic and Policlinic for Dermatology and Venereology, University Medical Center Rostock, 18057 Rostock, Germany; 3 Beijing Advanced Innovation Center for Soft Matter Science and Engineering, College of Life Science and Technology, Beijing University of Chemical Technology, Beijing 100013, China; 4 Department of Traumatology and Orthopedics, Peoples’ Friendship University of Russia, 117198 Moscow, Russia; 5 University Medical Centre, Justus Liebig University of Giessen, 35390 Giessen, Germany; 6 Department for Cell and Tissue Engineering, Scientific Research Center for Biomedicine, Faculty of Medicine, University of Niš, 18000 Niš, Serbia; 7 Department of Biology and Human Genetics, Faculty of Medicine, University of Niš, 18000 Niš, Serbia

**Keywords:** collagen membrane, barrier membrane, ossification, histomorphometry, correlation matrix analysis, M1-macrophages, M2-macrophages, bone tissue regeneration, in vivo, immunohistochemical staining

## Abstract

Barrier membranes are an essential tool in guided bone Regeneration (GBR), which have been widely presumed to have a bioactive effect that is beyond their occluding and space maintenance functionalities. A standardized calvaria implantation model was applied for 2, 8, and 16 weeks on Wistar rats to test the interactions between the barrier membrane and the underlying bone defects which were filled with bovine bone substitute materials (BSM). In an effort to understand the barrier membrane’s bioactivity, deeper histochemical analyses, as well as the immunohistochemical detection of macrophage subtypes (M1/M2) and vascular endothelial cells, were conducted and combined with histomorphometric and statistical approaches. The native collagen-based membrane was found to have ossified due to its potentially osteoconductive and osteogenic properties, forming a “bony shield” overlying the bone defects. Histomorphometrical evaluation revealed the resorption of the membranes and their substitution with bone matrix. The numbers of both M1- and M2-macrophages were significantly higher within the membrane compartments compared to the underlying bone defects. Thereby, M2-macrophages significantly dominated the tissue reaction within the membrane compartments. Statistically, a correlation between M2-macropahges and bone regeneration was only found at 2 weeks post implantationem, while the pro-inflammatory limb of the immune response correlated with the two processes at 8 weeks. Altogether, this study elaborates on the increasingly described correlations between barrier membranes and the underlying bone regeneration, which sheds a light on the understanding of the immunomodulatory features of biomaterials.

## 1. Introduction

Today, collagen membranes are integral components of the treatment concept of guided bone regeneration (GBR) for the augmentation of the jawbone or the healing of jaw ridge defects in combination with oral implant treatment in dentistry [1,2]. In recent decades, this material class has replaced non-degradable materials, such as polytetrafluoroethylene (PTFE)-based membranes, for most indications. This is mainly based on the fact that bioresorbable membranes prevent secondary surgery for removal. In this context, barrier functionality is still the main purpose of these medical devices in order to enable undisturbed bone-healing without potentially hindering effects outgoing from the soft tissue [3]. Thus, the application of an ideal barrier membrane must allow for the desired clinical regenerative outcome, which is bone regeneration for a following implant insertion. Interestingly, other (desired) requirements have been postulated in recent years, especially features of resorbable collagen-based barrier membranes alongside barrier functionality [3]. Among other things, a barrier membrane should “actively” support the process of bone-healing. In this context, a so-called “selective permeability” of the membrane must prevent the ingrowth of epithelial cells while promoting the proliferation of osteogenic cells. Furthermore, the optimal barrier membrane should allow the establishment of an adequate angiogenesis and vascularization for bone tissue regeneration in form of a so-called “transmembraneous vascularization” [4].

Additionally, different authors stated that the molecular micromilieu induced by a barrier membrane might also influence the molecular events of the neighboring bone tissue regeneration process [4,5,6,7]. Altogether, it has been assumed that tissue reactions to the membrane might have an active (molecular) biological role in the bone-healing process in addition to the bone substitute material (BSM) used. For example, the application of an ideal membrane could promote the recruitment and differentiation of different cell types that are involved in the bone-healing process, such as osteoblasts and precursor cells. Moreover, it has been assumed that the inflammatory tissue responses to a membrane could have a substantial influence on the underlying bone-healing process [8,9]. In this context, it has been suspected that biomaterials inducing an overall anti-inflammatory tissue reaction, including the respective macrophage subtype, i.e., anti-inflammatory M2-macrophages, may optimally contribute to the material-related tissue regeneration progress [10,11].

It has already been shown that biomaterials, including barrier membranes based on collagen from different animal and tissue sources, induce a higher anti-inflammatory cell responses, as demonstrated by the significantly higher numbers of M2-macrophages [6,7]. Specifically, a barrier membrane based on the porcine pericardium has been shown to fulfill the above-mentioned requirements as the device showed a mild and predominantly anti-inflammatory tissue reaction within the surrounding tissue and a required cell migration in combination with optimal barrier functionality without any signs of fragmentation for several months [12,13,14]. Additionally, it has been shown that this membrane allows for sufficient transmembraneous vascularization [4,5,14,15].

To test the hypothesis of the interaction between the bioactivity of barrier membranes and the underlying bone defects, the present study was conducted. The membrane was used in combination with a newly developed BSM (combining xenogeneic granules with hyaluronic acid) and implanted in Wistar rats at early (2 weeks), intermediate (8 weeks), and late (16 weeks) time points in a standardized calvaria implantation model [16]. As a control, a sham operation without BSM was performed at all time points. Thereby this implantation model was used to simulate the intraoral clinical indications that require GBR procedures, as the anatomical conditions (i.e., bone defects that are covered with soft tissue and epithelium) are comparable. Established histological evaluation methods in combination with histomorphometrical analyses were conducted to quantitatively examine the membrane degradation and new bone growth, as well as the immune response and vascularization [16,17]. The data were then employed in Pearson’s correlation analyses to further investigate the potential interactions between the membrane and the defect areas.

## 2. Results

### 2.1. Histological Results

**Figure 1:**
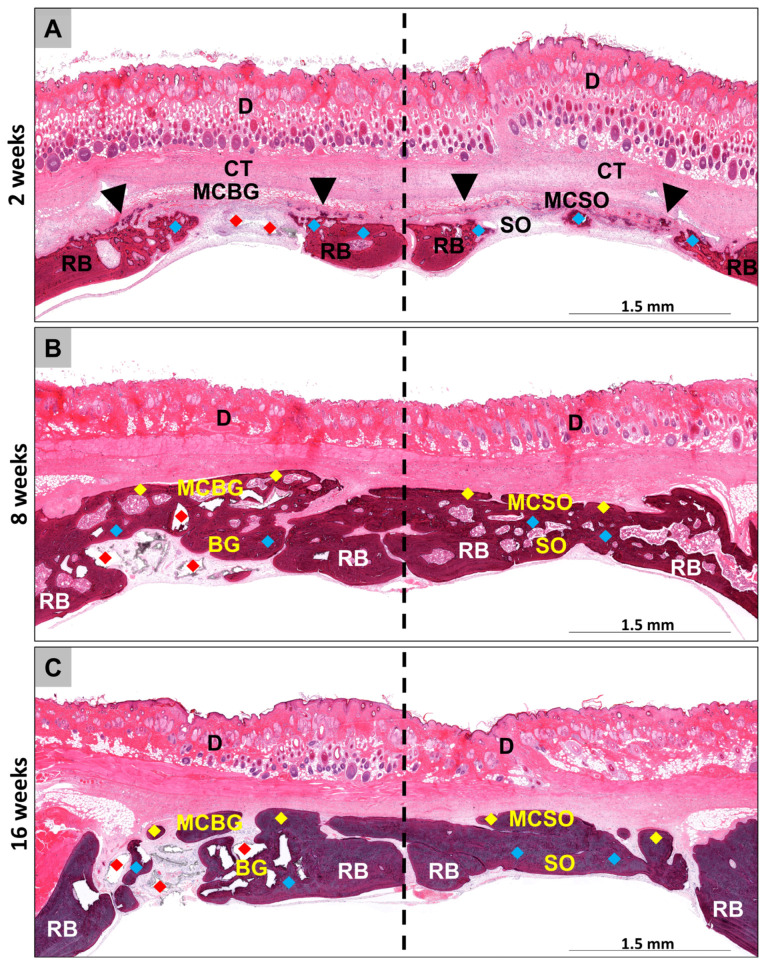
Overviews of the implantation beds at the three time points at (**A**) 2, (**B**) 8, and (**C**) 16 weeks post implantationem and the progression of the bone regeneration within the four compartments, i.e., the membrane compartment above bone graft-filled defects (MCBG), the bone graft-filled defects (BG), the membrane compartment above the sham operation defects (MCSO), and the sham bone defects (SO). blue rhombus = newly formed bone tissue; red rhombus = bone substitute material; black arrowheads = bone ingrowth into the membrane; yellow rhombus = ossified collagen membrane; RB = residual bone; CT = connective tissue; D = dermis (HE staining, 100× magnifications scale bars = 1.5 mm).

**Figure 2:**
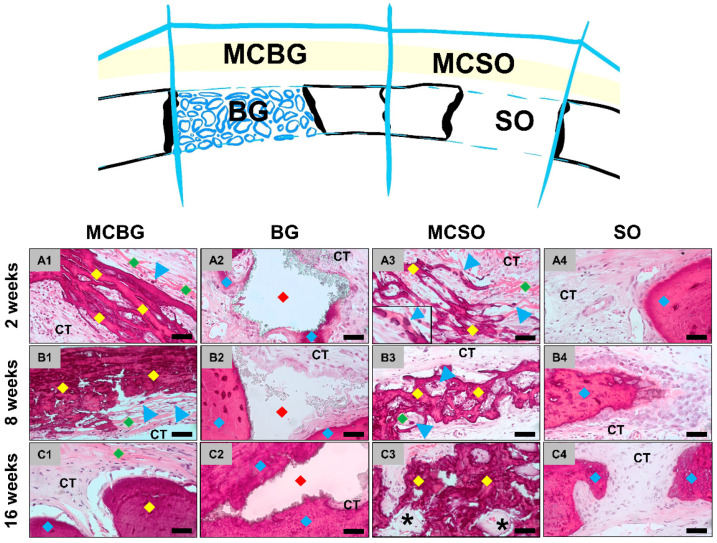
Histological images of the tissue responses in the four compartments at (**A1**–**A4**) 2, (**B1**–**B4**) 8, and (**C1**–**C4**) 16 weeks post implantationem. Left row: tissue responses within the membrane compartment above the bone graft-filled defect (MCBG). Left center row: tissue responses within the bone graft-filled defects (BG). Right center row: tissue responses within the membrane compartment above the sham operation defects (MCSO). Right row: tissue responses within the sham bone defects (SO). Green rhombus = collagen fibers of the membrane; blue arrowheads = ossification nuclei within the membrane fibers; yellow rhombus = ossified membrane (fibers); red rhombus = BSM granules; blue rhombus = newly formed bone with the bone graft-filled and sham operation defects; stars = areas of condensed connective tissue; CT = connective tissue (HE staining, 400× magnifications, scale bars = 20 µm).

At 2 weeks post implantationem, the histological analysis showed that the membrane was detectable and intact in both membrane compartments (Figure 1A). Moderate cellular infiltration into the membrane was observed (Figure 2(A1,A3)). Furthermore, a slight ingrowth of new bone matrix outgoingg from the bony defect borders was detectable in all compartments (Figure 1A and Figure 2(A1–A4)). An osteoconductive bone growth process was found in the group of the bone graft-filled compartments, while a mixture of osteoconductive and osteogenic bone growth was visible in both membrane compartments Figure 1A and Figure 2(A1–A4). Thus, an osteoconductive bone ingrowth extruding from the surrounding underlying bone tissue was visible, leading to the ossification of the membrane fibers, while independent ossification nuclei within the membrane fibers were also observable (Figure 2(A1,A3)). Thereby, a moderate inflammatory tissue response, mainly involving macrophages, alongside the lower numbers of granulocytes and lymphocytes in combination with a moderate implantation bed vascularization, was observed in all compartments. Furthermore, the low numbers of multinucleated giant cells (MNGCs) were detectable on the surfaces of the BSM granules.

The detection of M2-macrophages showed their occurrence within the membrane and within the intergranular connective tissue of the BSM granules and the surrounding tissue of both implant types (Figure S2). M2-macrophages were not seen directly on the surface of the collagen membrane or the BSM granules. The detection of M1-macrophages showed that in all compartments, this subtype was detectable at the surface of both biomaterials and within the membrane (Figure S1). Mono- and multinuclear cells expressing the CD11c molecule were also detected on the surfaces of the BSM.

Higher numbers of M1- and M2-macrophages were found in both membrane compartments than their vertically adjacent compartments. Moreover, comparable numbers of M1- and M2-macrophages were found in both membrane and bone defect compartments (Figures S1 and S2).

At 8 weeks post implantationem, a significant increase in newly formed bone matrix was seen in all compartments (Figure 1B and Figure 2(B1–B4)). In the implantation beds of the BSM and in the sham operation group, the central defect areas still contained connective tissue and thus showed no complete bone-healing, indicating a further osteoconductive bone growth process, extending from the defect borders towards the defect center (Figure 1B and Figure 2(B2,B4). In both membrane compartments, a further ossification of the collagen membrane took place up to a nearly complete calcification in some animals, building a “bony shield” above the bone defect areas, independent of the bone growth within the underlying compartments (Figure 1B and Figure 2(B1,B3)). Thereby, most of the collagen fibers of the membranes were calcified, while a few ossification nuclei within the membrane fibers were still observable, hinting at ongoing osteogenic bone growth (Figure 2(B1,B3)).

A slight inflammatory tissue response, mainly involving macrophages and low numbers of granulocytes, lymphocytes, and multinucleated giant cells (MNGCs) at the surfaces of the BSM granules in combination with moderate implantation bed vascularization, was observed in all compartments. At this study time point higher numbers of both macrophage subtypes were still found in both membrane compartments, compared to their vertically adjacent compartments. Comparable numbers of the M1- and M2-macrophages were found in both membrane compartments, as well as both bone defect compartments (Figures S1 and S2).

At 16 weeks post implantationem, a further increase in bone regeneration was seen in all compartments Figure 1C and Figure 2(C1–C4)). In the membrane compartments, only slight membrane remnants were detectable, while nearly the whole membranes were replaced by mature bone matrix, building a “bony shield” above the bone defect areas as mentioned above (Figure 1C and Figure 2(C1,C3)). In the membrane compartment above the sham operation, minor areas of the membrane were still filled by connective tissue that was condensed, hinting at a precursor matrix for the growth of bone matrix (Figure 2(C3)). At this study time point, a slight inflammatory tissue response was still present, mainly composed of macrophages in combination with low numbers of granulocytes, lymphocytes, and multinucleated giant cells (MNGCs) at the surfaces of the BSM granules, as well as a moderate implantation bed vascularization, which were detectable in the remaining areas of connective tissue in all compartments. At this late time point of study, higher numbers of M2-macrophages were found in both membrane compartments compared to the occurrence of M1-macrophages, while comparable numbers of M1- and M2-macrophages were found in defect compartments (Figures S1 and S2).

### 2.2. Histomorphometrical Results

#### 2.2.1. Bone Regeneration

**Figure 3:**
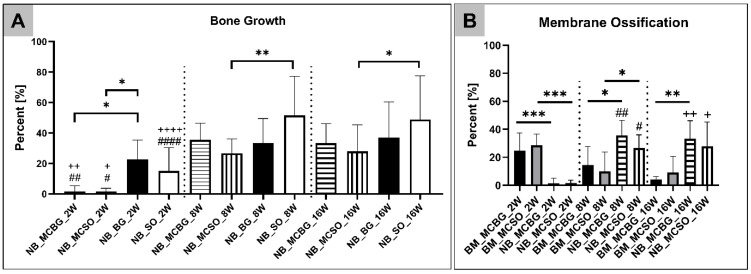
(**A**) Results of bone regeneration at 2, 8, and 16 weeks post implantationem. (**B**) Results of the membrane ossification measurements in comparison to membrane resorption within both membrane compartments. Intraindividual significances: * *p* < 0.05, ** *p* < 0.01, *** p < 0.001; interindividual significances: #/+ *p* < 0.05, ##/++ *p* < 0.01, and ####/++++ *p* < 0.0001. For (**A**,**B**), # indicates significant differences between the 2- and 8-week datasets and + indicates significant differences between the 2- to 16-week datasets. NB = newly formed bone; BM = biomaterial; MCBG: membrane compartment above bone graft-filled defects; MCSO: membrane compartment above sham operation; BG: bone graft-filled defect; and SO: sham operation bone defect.

At 2 weeks post implantationem, the measurements showed that in the groups of the bone graft-filled compartment and the sham operation, a comparable bone regeneration was found (Table 1 and Figure 3A). Additionally, in both membrane compartments, a slightly comparable bone ingrowth was detectable (Table 1 and Figure 3A). The analysis furthermore showed that only a significant difference (* *p* < 0.05) was found between the bone regeneration values in the bone graft-filled compartment and in both membrane compartments (Table 1 and Figure 3A). At 8 and 16 weeks post implantationem, the analysis revealed that comparable bone regeneration values in the different compartments were detectable with the exception of a significant difference (** *p* < 0.01 and * *p* < 0.05) between the amount of newly formed bone matrix in the groups of the sham operation and its related membrane compartment (Table 1 and Figure 3A).

Additionally, the measurements of the new bone within the membrane compartments revealed that a significantly higher comparable percentage (*** *p* < 0.001) of the membranes in both compartments was identifiable as only a slight comparable ossification fraction was observed at 2 weeks post implantationem (Table 1 and Figure 3B).

At 8 weeks post implantationem, the significant percentages (* *p* < 0.05) of the membranes in both compartments were ossified (Table 1 and Figure 3B). The percentages of membrane ossification were also significantly higher (# *p* < 0.05 and ## *p* < 0.01) compared to the respective values at 2 weeks post implantationem (Table 1 and Figure 3B).

At 16 weeks post implantationem only in the group of the membrane compartment above the bone graft-filled bone defects a significantly higher membrane ossification (** *p* < 0.01) was detected (Table 1 and Figure 3B). Moreover, the ossification values at this time point were significantly higher (+ *p* < 0.05 and ++ *p* < 0.01) compared to the values at 2 weeks post implantationem (Figure 3B).

An exemplary histological image, showing the transition of collagen to bony tissue is found in the Supplementary Materials (Figure S4).

#### 2.2.2. Immune Response

**Figure 4:**
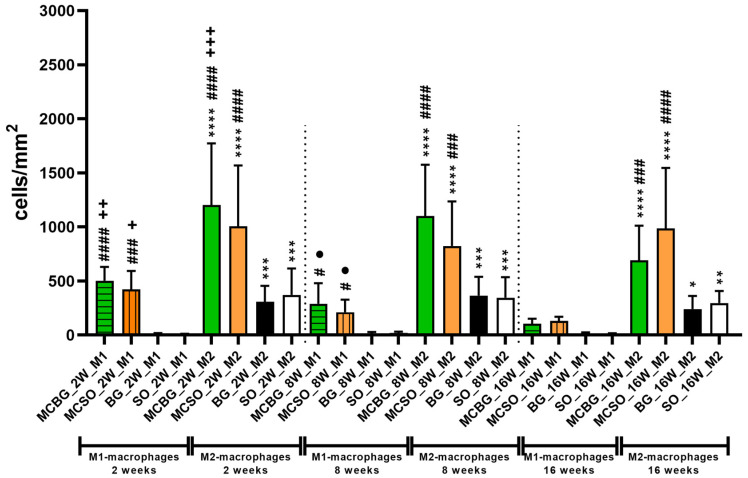
Results of the macrophage subtype measurements at 2, 8, and 16 weeks post implanta-tionem. Intraindividual significant differences: * *p* < 0.05, ** *p* < 0.01, *** *p* < 0.001, and **** *p* < 0.0001. Interindividual significant differences: # *p* < 0.05 ### *p* < 0.001, and #### *p* < 0.0001, + *p* < 0.05, ++ *p* < 0.01, and +++ *p* < 0.001. + = significant differences between 2- and 16-week datasets; ● = significant differences between 8- and 16-week datasets. MCBG: membrane compartment above bone graft-filled defects; MCSO: membrane compartment above sham operation; BG: bone graft-filled defect; and SO: sham operation bone defect.

The histomorphometrical analysis of the occurrence of pro- and anti-inflammatory macrophage subtypes within the four compartments initially revealed that significantly higher numbers of M2-macrophages (* *p* < 0.05, ** *p* < 0.01, *** *p* < 0.001, and **** *p* < 0.0001) were detectable in all compartments and at all three time points (Table 2 and Figure 4). Furthermore, the analysis showed that the numbers of pro- and anti-inflammatory macrophages were significantly higher (# *p* < 0.05, ### *p* < 0.001, and #### *p* < 0.0001) within both membrane compartments, i.e., the MCBG and the MCSO compartments, compared to the respective numbers within the bone defect compartments, i.e., the BG and the SO compartments at 2 and 8 weeks post implantationem (Table 2 and Figure 4). At 16 weeks post implantationem no significant differences between the numbers of M1-macrophages were detectable in all four compartments, while significantly higher numbers of M2-macrophages (### *p* < 0.001 and #### *p* < 0.0001) were found within both membrane compartments compared to both bone defect compartments (Table 2 and Figure 4).

Additionally, the analysis revealed that a significant decrease in the M1-macrophage numbers in the MCBG and the MCSO compartments and of the M2-macrophages within the MCBG compartment was detectable between 2 and 16 weeks post implantationem (+ *p* < 0.05, ++ *p* < 0.01, and +++ *p* < 0.001) (Figure 4). Additionally, a significant decrease in the M1-macrophage numbers was observed in the MCBG and the MCSO compartments between 8 and 16 weeks post implantationem (*p* < 0.05) (Figure 4).

#### 2.2.3. Vascularization

**Figure 5:**
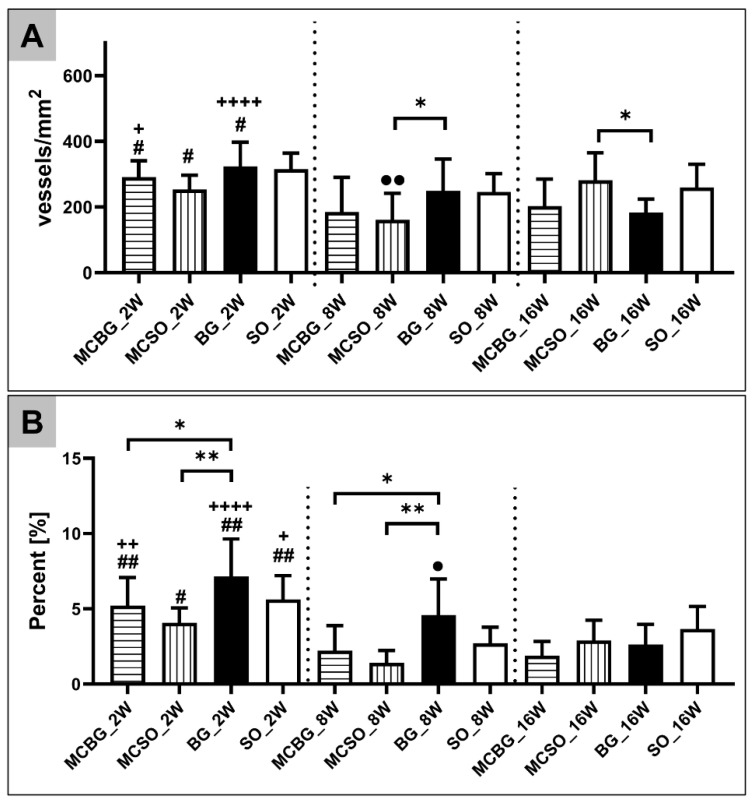
Results of the measurements of (**A**) the vessel density and (**B**) the vascularization percentage at 2, 8, and 16 weeks post implantationem. Intraindividual significant differences are indicated as * *p* < 0.05 and ** *p* < 0.01. Interindividual significant differences are indicated as #/+/• *p* < 0.05, ##/++/•• *p* < 0.01, and ++++ *p* < 0.0001. # = significant differences between 2- and 8-week datasets; + = significant differences between 2- and 16-week datasets; • = significant differences between 8- and 16-week datasets.

The histomorphometrical analysis of the implantation bed vascularization showed that comparable vessel density values were detected in all compartments at 2 weeks post implantationem (Table 3 and Figure 5A). At 8 and 16 weeks post implantationem, only a significant difference (* *p* < 0.05) between the vessel densities within the MCSO compartment and the BG compartment was found (Figure 5A). Furthermore, the statistical analysis revealed that significant decreases in the vessel densities (# *p* < 0.05) in all compartments, with the exception of the SO compartment between 2 and 8 weeks post implantationem, were measured (Figure 5A). Additionally, significant decreases in the vessel densities (+ *p* < 0.05 and ++++ *p* < 0.0001) in the MCBG compartment and the BG compartment between 2 and 16 weeks post implantationem were found (Figure 5A). Finally, a significant decrease in the vessel density (*p* < 0.01) in the MCSO compartment between 2 and 16 weeks post implantationem was found (Figure 5A).

The analysis of the vascularization percentage, relevant to the TIA, showed that the highest values were found in the BG compartment at 2 and 8 weeks post implantationem and that were significantly higher (* *p* < 0.05 and ** *p* < 0.01) than the values in the MCBG and the MCSO compartments, while no difference was found compared to the SO compartment (Table 3 and Figure 5B). Furthermore, the statistical analysis revealed that significant decreases in the vascularization percentage (# *p* < 0.05 and ## *p* < 0.01) in all compartments between 2 and 8 weeks post implantationem were measured (Figure 5B). Additionally, significant decreases in the vascularization percentages at 2 weeks (+ *p* < 0.05, ++ *p* < 0.01, and ++++ *p* < 0.0001) in the MCBG compartment, the BG compartment, and the SO compartment were detected, compared to 16 weeks (Figure 5B). Finally, a significant decrease in the vascularization percentage (*p* < 0.05) in the BG compartment between 8 and 16 weeks post implantationem was measured (Figure 5B). The histological figures of the immunohistochemical detection of vascularization are provided in the Supplementary Materials (Figure S3).

### 2.3. Correlation Analyses

**Figure 6:**
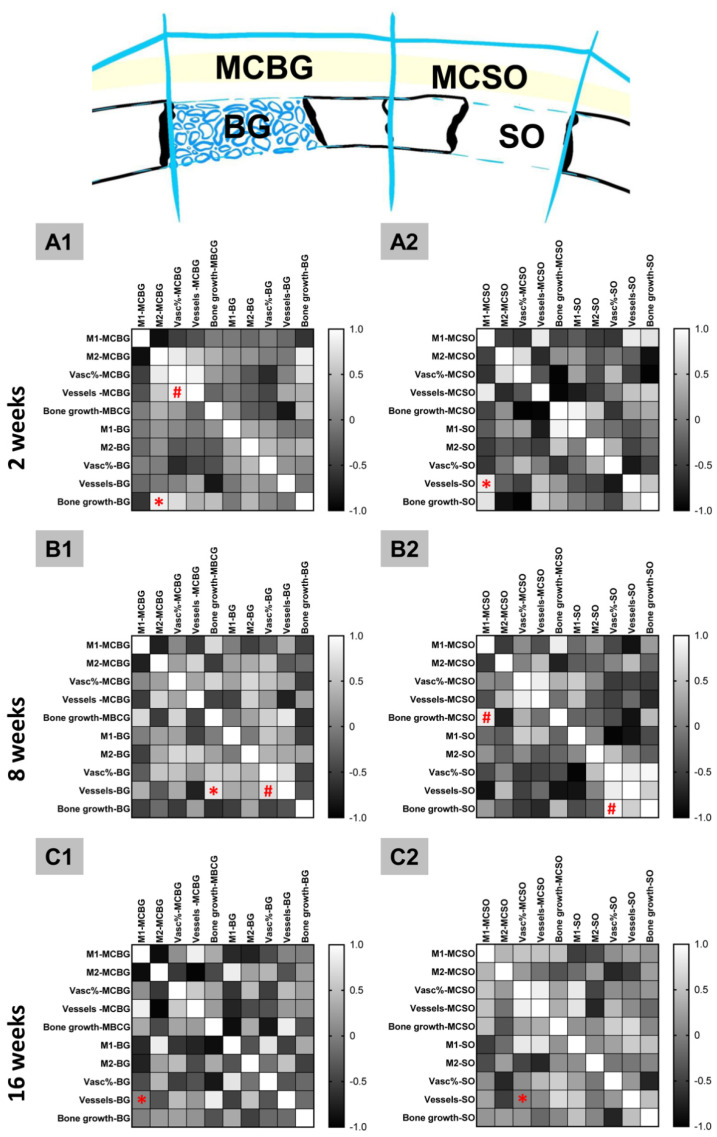
Correlation matrices between all measured variables within and between overlying compartments, i.e., the MCBG and the BG compartments (left row, **A1**–**C1**) and the MCSO and the SO compartments (right row, **A2**–**C2**) at 2, 8, and 16 weeks post implantationem. Asterisks = sig-nificances between compartments; hashtags = significances within a compartment. MCBG: mem-brane compartment above bone graft-filled defects; MCSO: membrane compartment above sham operation; BG: bone graft-filled defect; and SO: sham operation bone defect.

Pearson’s correlation analysis was conducted to explore positive correlations between two measured parameters within the same compartment or between overlying coherent compartments, i.e., the MCBG and the BG compartments or the MCSO and the SO compartments (Figure 6).

At 2 weeks post implantationem, a positive correlation (*p* = 0.023) between the bone growth in the BG compartment and the occurrence of M2-macrophages in the MCBG compartment was found (Figure 6). Additionally, a positive significant correlation (*p* = 0.033) between the vessel density and the vascularization percent within the MCBG compartment was calculated (Figure 6(A1)). Moreover, a positive correlation (*p* = 0.041) between the vessel density in the SO compartment and the occurrence of M1-macrophages within the MCSO compartment was measured (Figure 6(A2)).

At 8 weeks post implantationem, a positive correlation (*p* = 0.009) between the vessel density in the BG compartment and the bone growth within the MBCG compartment was measured (Figure 6(B1)). Additionally, a positive correlation (*p* = 0.013) between the vessel density and the vascularization percentage in the BG compartment was found (Figure 6(B1)). Furthermore, a positive correlation (*p* = 0.017) between the bone growth within the MCSO compartment and the occurrence of M1-macrophages within the same compartment was found (Figure 6(B2)). Finally, a positive correlation (*p* = 0.018) between the bone growth and the vascularization percent within the SO compartment was revealed (Figure 6(B2)).

At 16 weeks post implantationem, a positive correlation (*p* = 0.021) between the vessel density in the BG compartment and the occurrence of M1-macrophages in the MCBG compartment was calculated (Figure 6(C1)). Finally, a positive correlation (*p* = 0.022) between the vessel density in the SO compartment and the vascularization percent in the MCSO compartment was revealed at this time point (Figure 6(C2)).

## 3. Discussion

The aim of the present study was to analyze the molecular interactions between the bioactivity of collagen-based barrier membranes and the underlying bone defects. To achieve this, a pericardium-based collagen membrane was used in combination with a newly developed BSM combining xenogeneic granules with hyaluronic acid in an established calvaria implantation model in Wistar rats for 2, 8, and 16 weeks post implantationem [16]. This BSM, mixed with hyaluronic acid as a water-binding molecule, was investigated in a previous study by our group [16]. The addition of hyaluronic acid did not yield in any differences regarding immune response, vascularization, and bone growth but improved clinical handling; therefore, only BSM mixed with hyaluronic acid was used in this study. This implantation model was conducted to simulate the intraoral clinical indications that require GBR procedures as the anatomical conditions (i.e., bone defects that were covered with soft tissue and epithelium) are comparable.

Initially, both the histological and the histomorphometrical analyses revealed that a gradual ossification within both the two bone defect compartments (filled with the bone substitute material as well as in the control compartment) and within the two membrane compartments was detectable. Only at 2 weeks post implantationem, a higher osteoconductive bone growth was found, in the bone defects compartment filled with the BSM especially, while only a very slight bone regeneration took place in both membrane compartments. However, starting from 8 weeks and up to 16 weeks post implantationem, comparable bone regeneration values were detected in all four compartments. Thereby, no signs of resorption of the xenogeneic bone substitute granules were observable, as had also been previously described in the case of this material class [18,19,20]; however, the collagen membrane was gradually resorbed while being completely transformed into bone tissue or bone matrix based on its fiber structure. In this context, the ossified membrane formed an independent bony layer above the actual bone defect areas. The gradual bone growth around the BSM is expected as such materials have been reported to enhance bone growth in such a fashion [18,21,22]. However, the bone transversion of the “native” collagen membrane is a surprising result as this phenomenon has only been described in a very small number of studies [23,24,25]. For example, in a preclinical study, Taguchi et al. described the ossification of a native bi-layered collagen membrane [25]. The authors concluded that the collagen matrix allows for bone growth, serving as both an osteoconductive and an osteogenic scaffold. Comparable to the results of the present study, it was observed that new bone formation within the membrane was independent of regeneration from the bone defect cavity. Moreover, it was described that the collagen matrix induced osteoblastic differentiation as also described in the case of the pericardium-based collagen membrane analyzed in the present study showing “ossification nuclei” with associated active osteoblasts and matrix deposition along the collagen fiber bundles, which is similar in definition to the physiological endochondral bone growth. Additionally, in both studies, it was not possible to distinguish the new membrane-derived bone matrix from that which was outgoing (via osteoconduction) from the residual bone tissue. In a previous study, it was concluded that the osteogenic bone growth was driven by mediating factors, such as bone morphogenetic proteins, transforming growth factors, insulin-like growth factors, and fibroblast growth factors, that might have (indirectly) bound to the collagen fibers [25]. In another study conducted by Zubery et al., it was demonstrated that a cross-linked porcine collagen barrier membrane also ossified with time and augmented the original alveolar ridge in both preclinical and clinical studies [23,24]. In the case of this membrane, which is cross-linked via ribose, only osteoconductive bone growth was observed in areas of direct contact with residual bone indicating that this membrane did not induce both regeneration pathways as described above. Interestingly, the above-mentioned non-cross-linked bi-layered type I and III porcine collagen membrane, which was used as control material in both studies, showed very few signs of membrane ossification. The results of the present study revealed that the analyzed pericardium-based membrane did allow for a combination of osteoconductive and osteogenic bone regeneration and did contribute to a faster and complete bony substitution within both membrane compartments compared to the previously described studies. It can also be concluded that the ossification and the associated generation of the described “bony shield” might help to regenerate the underlying bone defect by means of additional bone ingrowth and due to the protection of the underlying defect area. However, further preclinical trials, including large animal models and appropriate clinical trial settings, need to be conducted to verify these observations.

In addition to their osteoconductive properties, it has been proposed by different authors that the molecular micromilieu induced by biomaterials in general and thus by a barrier membrane might also influence the molecular events of the neighboring bone tissue regeneration process [26,27,28,29]. In this context, it has widely been assumed that the inflammatory tissue responses to a membrane and especially an overall anti-inflammatory tissue reaction, involving mainly M2-macrophages, might have a substantial influence on the underlying bone-healing process [30,31]. Moreover, it has been stated by different authors that the optimal barrier membrane allows for sufficient transmembraneous vascularization to contribute to bone regeneration [4,5,15]. However, there have been no studies to date that examine these molecular relationships. Based on study protocols that have already been published in various preliminary studies, the present study was aimed directly at this analysis [7,16,32]. In this context, the histomorphometrical analyses of the vascularization pattern and the occurrence of pro- and anti-inflammatory macrophage subtypes were conducted to correlate both parameters with the bone regeneration process. The analysis of the vascularization within the four different compartments revealed that comparable vessel densities and vascularization percentages were found within all compartments over the complete study period except for the significantly higher values of both parameters in the bone graft-filled bone defect compartment. This latter result can be explained by the higher number of multinucleated giant cells that are known to be potent sources of proangiogenic factors, such as the vascular endothelial growth factor, (VEGF) and are particularly induced in the case of bone substitute materials, such as the xenogeneic material used in the present study [33].

Furthermore, the analysis of the macrophage subtypes showed that within the four compartments significantly higher numbers of M2-macrophages were initially detectable in all compartments and at all three time points. These results show that the used biomaterials, i.e., the bone substitute material as well as the collagen membrane, can be classified as biocompatible, as they did not induce exaggerated pro-inflammatory tissue reactions. Furthermore, this result is in line with the assumption of the immunocompatibility of biomaterials, as it was stated that an optimal material should induce an overall anti-inflammatory tissue reaction and macrophage response to support (bone) tissue regeneration [34]. Additionally, the comparable values in the material compartments to the macrophage numbers within the SO compartment are noticeable and this result additionally reveals that both biomaterials, i.e., the BSM and the collagen membrane, do not induce adverse cell reaction patterns or pro-inflammatory tissue responses beyond the “basal” level, i.e., a reaction that does not exceed that usually involved in the wound-healing process.

Additionally, the numbers of pro- and anti-inflammatory macrophages were significantly higher within both membrane compartments compared to the respective numbers within the bone defect compartments. Only at 16 weeks post implantationem were no significant differences between the numbers of M1-macrophages detectable in all four compartments, while significantly higher numbers of M2-macrophages were found within both membrane compartments compared to both bone defect compartments. This measurement result can be explainable on the basis of the biodegradation patterns of both biomaterials used in the present study. Thus, the membrane has been shown to degrade faster even in comparison to the xenogeneic BSM granules. In this context, it has already been shown that the integration and the associated biodegradation of collagen membranes are processed mainly by macrophages, which was confirmed by this study result once again [4,7]. Therefore, faster biodegradation is also accompanied by the migration of a higher number of phagocytes, such as the measured macrophages. Since the membrane was almost completely ossified after 16 weeks and also integrated or degraded, it can be assumed that the further migration of phagocytosing macrophages was no longer “required” to be induced.

Finally, the analyses of positive correlations revealed two different “dependency categories”, i.e., (a) correlations between tissue parameters within the same compartment and (b) between correlating parameters within vertically adjacent compartments (bone defect compartment and neighbored membrane compartment).

At 2 and 8 weeks post implantationem, correlations between the vessel density and the vascularization percent within the MCBG compartment and the BG compartment were found, respectively. This result is not surprising because it has already been proven that the cell types involved in the (inflammatory) tissue responses to biomaterials, such as the used bone substitute material and the pericardium-based barrier membrane (mainly macrophages and MNGCs), express different mediating factors, such as different members of the VEGF family [5,33,35,36]. The different VEGF isoforms have been shown to induce various signaling pathways, also including angiogenesis and vessel maturation, so that a connection to this signaling family is conceivable [37,38]. Additionally, the combinatory effects of two or more different mediators, such as the fibroblast growth factor 2 (FGF2) and VEGF, have been shown to increase both angiogenesis and blood vessel maturation when combining both mediators with collagen–heparin scaffolds [38]. Additionally, vascular pericytes and mural cells support cells that range in phenotype from pericytes to vascular, and smooth muscle cells have been shown to be involved in the process of vessel maturation [39].

Bone growth and the vascularization percentage within the SO compartment were also found to correlate at 8 weeks post implantationem. This result was also predictable as both direct and indirect interactions between vascularization pathways and the molecular processes of bone regeneration have been identified [40,41]. The achievement of bone tissue regeneration is focused on the primary role of vascularization occurrence in order to deliver enough nutrients, growth factors, minerals, oxygen, and relevant cells for tissue restoration [42]. Additionally, in this context, VEGF has been shown to indirectly stimulate osteogenesis [40].

Moreover, a correlation between the bone growth process and the occurrence of M1-macrophages within the MCSO compartment was detectable at 8 weeks post implantationem. This result appears initially surprising, as it has widely been stated that biomaterials that induce a predominantly anti-inflammatory tissue response are also likely to improve (bone) tissue regeneration [8,43]. However, this correlation might also be explainable on the basis of vascularization patterns. In this context, it is well known that every biomaterial induces a material-specific inflammatory tissue response [44]. Even bioresorbable materials, as used in the present study, provoke granulation tissue, including phagocytes containing macrophages and partially containing multinucleated giant cells (MNGCs) that are involved in biodegradation [7,16]. A vascularization pattern dependent on the rate of biodegradation and associated inflammation is also an essential component of this granulation tissue—if only to guarantee the movement and clearance of phagocytes and their progenitor cells [45]. Additionally, it has been revealed by Abels et al. that the key role of the proinflammatory M1-macrophages is the phagocytic activity towards a hydroxyapatite-based BSM, together with other studies that showed the same phenomenon in the case of collagen-based biomaterials [46]. The histomorphometrical data of the afore-mentioned study about BSM indicate that the small BSM granules underwent a higher biodegradation—although no differences in the overall M1- or M2-macrophage numbers compared to the other groups were measured. Transferred to the results of the present study, which showed a correlation between the occurrence of M1-macrophages and the bone growth process within the membrane compartment of the sham operation group, this result might indicate that the biodegradation via membrane-induced M1-macrophages is linked with an associated vascularization pattern, which might have supported the bone regeneration process. The question of why this phenomenon was not detectable within the membrane compartment neighbored to the bone graft-filled bone defects arises and an answer to this may be found in the interactions between the membrane and bone defect areas, which are discussed below.

Initially, this analysis part showed that a correlation between the bone growth in the BG compartment and the numbers of M2-macrophages in the related MCBG compartment were detectable at 2 weeks post implantationem. This result is in line with the results of other studies that describe the regenerative role of this subtype in (bone) tissue regeneration [30,31]. In the context of bone regeneration, different pathways, e.g., the regulation of mesenchymal stem cell and osteoblast function, based on factors such as macrophage-derived microRNAs (miRNAs), have been described as contributing to positive or negative regulation [47]. However, it is surprising that this observation was made only at this early time point, since it is assumed that a switch from M1-macrophages to M2-macrophages also occurs only later. However, this can be explained by the general decrease in macrophage numbers measured in the present study.

Furthermore, the vessel density in the SO compartment and the occurrence of M1-macrophages within the associated MCSO membrane compartment correlated at this early time point. Interestingly, the same correlation was found at 16 weeks post implantationem, as the vessel density within the BG compartment and the occurrence of M1-macrophages within the MCBG compartment were shown to be significantly linked. As mentioned above, it might be that the membrane-induced M1-macrophages also influenced the angiogenesis process in the associated bone defect area.

Additionally, the vessel density within the BG compartment and the bone growth within the MCBG compartment correlated at 8 weeks post implantationem. On the one hand, this result is interesting, since the diffusion distance for nutrient transport (oxygen: ~50 µm, nutrients: ~200 µm) is restricted [48,49]. This means that cells that are further away from an implantation bed of a membrane or the BSM undergoes nutrient limitations, which should lead to non-optimal conditions for new bone matrix production on the basis of interactions between both compartments. Thus, a direct influence seems to be only conditionally presumable, while it is conceivable that even indirect pathways between vascularization and bone growth in both compartments might be linked. For example, it has been reported that sufficient vascularization also leads to the improved transport of progenitor cells such as pre-osteoblasts [41]. Additionally, the relevant cells are capable of local migration or “crawling” via chemotaxis [50]. In this way, a correlation between the two parameters in the two compartments can probably also be explained as an underlying factor of osteoconductive bone growth from one compartment to the neighboring implantation area.

Finally, a correlation between the vessel density within the SO compartment and vascularization percent within the MCSO compartment was measured at 16 weeks post implantationem. On the one hand, it is conceivable that direct interactions between the implantation sites of both compartments also took place. However, it is also more likely in this case that indirect factors contributed to this correlation due to the distances between the two compartments. In this context, is has been described that vessel maturation, which means a higher vascularization percentage in contrast to a higher vessel density formed by vasculogenesis, requires not only the recruitment of mural cells but also support from a sufficient extracellular matrix [51]. Thus, the ongoing bone growth and bone-healing process in both compartments might be an explanation for this correlation. In this context, the blood vessel maturation might be controlled by osteoblasts (and other related cell types) based on a negative feedback loop mediated by the VEGF-induced secretion of the notch-1 receptor ligand, delta-like 4 (DLL4) [52]. An enhanced DLL4 expression downregulates the expression of VEGFR2 and thus EC proliferation. Additionally, the different VEGF isoforms secreted by osteoblasts amongst other cell types, such as VEGF121 and VEGF165, might have contributed to these measurement results [40]. In addition, the described transmembraneous vascularization of the pericardium-based barrier membrane might be an underlying factor for vessel maturation as endothelial cells have shown to trigger vessel maturation via the recruitment of pericytes to the newly synthesized branches via platelet-derived growth factor-BB (PDGF-BB) expression [53]. This is described by strictly imagining that the overlying compartments are physically separated, but in reality, these vertically adjacent compartments share an intertwined and complex vascular network.

Altogether, the present study substantiates in more detail the already widely suspected correlations between the two classes of materials [3,27,54]. Thereby, the correlations also show clear interactions between the pro- and anti-inflammatory immune responses to the biomaterials as well as the processes of implant bed vascularization and material-supported bone growth. Interestingly, the influence of M2-macropahges was only found at 2 weeks post implantationem, while the pro-inflammatory limb of the immune response correlated with the two processes at 8 weeks. These results are shedding new light on the widely described development of immunomodulatory materials. This suggests that while (bone) tissue regeneration requires typical regenerative features (i.e., biocompatibility, vascularization, etc.), it should be seen as a complex process that can be modulated (and even enhanced) by the optimal balance of the immune response. Furthermore, the result substantiates the direct correlation of the vascularization process with bone tissue regeneration—also in the context of the biomaterial application. The ossification of the collagen membrane represents another crucial observation. On the one hand, this demonstrates the bone-healing-promoting effect of long-term stable collagen materials, such as the pericardium-based membrane studied here. Furthermore, it can be assumed that the ossification of the membrane also leads to significantly better bone-healing and bone defect-shielding and stability after its clinical application.

## 4. Materials and Methods

### 4.1. Biomaterials

#### 4.1.1. Collagen-Based Barrier Membrane

The pericardium-based collagen membrane (Jason^®^ membrane, Botiss Biomaterials GmbH, Zossen, Germany) was used in the present study to cover the bone defects. This membrane was obtained from porcine pericardium. The medical device was prepared by means of a standardized manufacturing process that begins with an initial selection process of the donor animals and was followed by a purification process, including a wet-chemical treatment and lyophilization finalized by ethylene oxide gas sterilization. The collagen membrane is composed of a natural, multilayered structure with grid-like collagen fibers. Microscopically, the membrane has a honeycomb-like porosity. The biocompatibility and clinical usability of the membrane have been tested in different studies [7,15,55,56]. For the purpose of this study, the membrane (20 × 30 mm^2^) was cut into four equal rectangular parts. The membrane has been reported to have thickness range of 0.05–0.35 mm [57].

#### 4.1.2. Xenogeneic Bone Substitute Material

The bone substitute material (BSM) cerabone^®^ plus (Botiss Biomaterials GmbH, Zossen, Germany) combines the established bovine bone graft substitute cerabone^®^ with hyaluronic acid (high molecular weight), resulting in “sticky bone” BSM after hydrogenation [16]. The xenogeneic BSM cerabone^®^ was harvested from the femoral heads of cattle from registered slaughterhouses in New Zealand and Germany. The potentially immunogenic components were removed in a multi-step process to ensure their safe application [58]. In this process, the bovine bone raw material undergoes an ensured three-step heating, which is free of chemical additives and includes high-temperature treatment at over 1200 °C. cerabone^®^ plus must be hydrated before application (~0.5 mL saline solution per 1 mL cerabone^®^ plus) in order to result in a malleable bone grafting material, which facilitates the application and reduces particle distribution in the augmentation area. For the present study, cerabone^®^ plus with a granule size of 0.5–1 mm was used. Further chemical and structural analyses have been previously reported [59].

### 4.2. In Vivo Study

#### 4.2.1. Experimental Design

The calvaria implantation model was used to simulate the anatomical conditions of GBR procedures, i.e., bone defects with adjacent soft tissue and epithelium. Thereby, the implantations including the creation of defects within the calvaria were performed as previously described [16]. The study included 21 (N = 7) male 10–12-week-old male Wistar rats with a mean weight of 230 ± 20 g. Prior to the implantation procedure, the experimental animals were randomly divided into three different study groups for the respective time points, i.e., 2, 8, and 16 weeks post implantationem.

Prior to the study, the in vivo experiments were authorized by the local Ethical Committee of the Faculty of Medicine (University of Niš, Serbia) based on the approval number 323-07-00073/2017-05/7 of the Veterinary Directorate of the Ministry of Agriculture, Forestry and Water Management of the Republic of Serbia (date of approval: 22 February 2017).

The calvaria implantation procedure was performed in a standardized manner in accordance with the DIN ISO norm 10993-6 [60]. After initial anesthesia by means of an intraperitoneal injection with ketamine (100 mg/kg of body weight) and xylazine (5 mg/kg of body weight), the process was as follows: the shaving and disinfection of the skull region, the sagittal incision into the skin, the stripping of the periosteum, and the induction of bilateral cranial defects with a respective diameter of 4 mm with a trephine bur (GC, Tokyo, Japan). All left-sided defects were treated with equal amounts of the BSM, while the right-sided defects were treated as controls without material implantation. Prior to suturing, the defects were covered with the pericardium-based collagen membrane. The animals were routinely checked on and their health was documented. Water and food were available ad libitum until the designated time point. After the respective healing periods, the animals were euthanized using euthasol (400 mg/mL), the defect sites were removed and transferred to 4% formalin solution. Afterwards, routine histological evaluation was performed.

#### 4.2.2. Histological Evaluation

The explants were embedded in the polymer embedding system Technovit 9100 (Technovit 9100, Kulzer GmbH, Hanau, Germany). After initial dehydration, stepwise immersion at 4 °C with Technovit 9100 medium using specific infiltration solutions (following the manufacturer’s instructions) was conducted. Afterward, the tissue blocks were trimmed into a diamond shape using a grinding machine (EcoMet 30, Bühler, Esslingen, Germany) and sectioned at 7 µm using a specialized rotation microtome for hard tissue samples (CUT4060E, microTec GmbH, Walldorf, Germany). Finally, the slides were histochemically stained with hematoxylin and eosin (H&E) and Movat Pentachrome staining. The immunohistochemical staining of the transmembrane expression of CD11c (abx231412, Abbexa Ltd., Milton, UK) and CD163 (ab182422, Abcam, Cambridge, UK) was used for the detection of M1- and M2-like macrophages, respectively. CD31 antibody (ab182981 Abcam, Cambridge, UK) was used for the detection of vascular endothelial cells. The process of immunohistochemical staining has been thoroughly described by Lindner et al. [32]. The antigen retrieval sections were (pre-)treated with TRIS-EDTA pH 9 for 20 min at 96 °C to recover antigen reactivity. To prevent non-specific binding, the sections were incubated with a blocking solution for 10 min. Primary antibodies (CD11c, CD163, and CD31) were incubated at room temperature for 60 min, 40 min, and overnight, respectively. Afterward, the biotinylated secondary antibody was applied to bind several molecules of the permanent streptavidin-alkaline phosphatase (AP)-red conjugate. Upon the addition of the chromogenic solution, the target antigen was detected via a red-pink reaction product. Unless otherwise stated, all solutions and reagents for immunohistochemical analysis were purchased from Zytomed Systems (Berlin, Germany).

#### 4.2.3. Histological Analyses

Histological analysis was performed using an Axioscope 5 microscope (Zeiss, Oberkochen, Germany). The analyses included the observations of the tissue-biomaterial interactions, including the cell reactions as well as tissue responses involving (bone) tissue integration and vascularization. Microphotographs were taken using a scanning microscope (M8, PreciPoint, Munich, Germany).

#### 4.2.4. Histomorphometry

**Figure 7:**
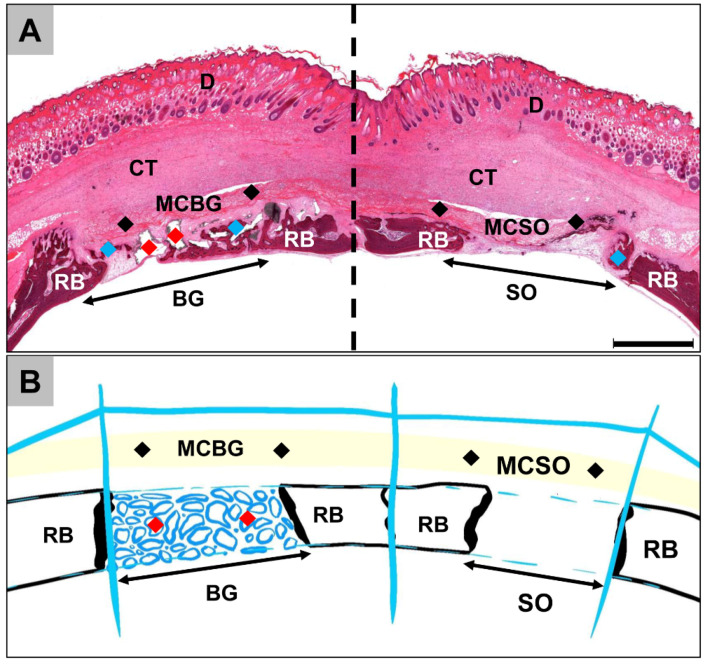
(**A**,**B**) Schematic overview of a calvaria, involving the four different compartments used for histomorphometrical measurement. BG and double-arrow = bone graft-filled bone defect; SO and double-arrow = sham-operated bone defect; MCBG = membrane compartment above bone graft-filled bone defect; MCSO = membrane compartment above sham defects; blue rhombus = newly formed bone tissue; red rhombus = bone substitute material; black rhombus = collagen membrane; RB = residual bone; CT = connective tissue; D = dermis. (**A**) HE staining, “total scan”, 100× magnification, scale bar = 5 mm.

To enable histomorphometry, total scans were generated using a scanning microscope (M8, PreciPoint, Munich, Germany). The histomorphometry was achieved by means of image analyses via the software ImageJ v 1.53 (National Institutes of Health, Bethesda, MD, USA) [32]. To determine the different parameters within the specific areas of the implantation beds, the margins were manually integrated to separate the different compartments after determining the total implantation area (TIA) (Figure 7A,B):

i Membrane compartment above bone graft-filled bone defects (MCBG);
ii Membrane compartment above sham defects (MCSO);
iii Bone graft-filled bone defects (BG);
iv Sham bone defects (SO).

The four compartments were then utilized to measure the respective areas of newly formed bone, remaining membrane, remaining BSM, and connective tissue. Percentages of those areas were calculated with respect to the areas of the four compartments. Moreover, the occurrence of the M1- and M2-macrophage subtypes, as well as the vascularization pattern, were analyzed using a specialized plugin on ImageJ that was developed by Lindner et al. [32,61]. This plugin allows us to measure the number of stained cells per mm^2^ and the vascularization pattern, i.e., the vessel density (vessels/mm^2^) and vascularization percent (percentage of vessel areas related to the TIA). Briefly, the detection of stained areas was carried out via ImageJ, whereas vessel density was the measurement of the number of detected vessels over the assigned area. Furthermore, the percentage of vascularization was measured by measuring the area of detected vessel and dividing it over the assigned area.

### 4.3. Statistical Analyses

The quantitative data were initially confirmed and approved for normality via the D’Agostino–Pearson and Shapiro–Wilk normality tests by means of the GraphPad Prism 9.3.1 software (GraphPad Inc., La Jolla, CA, USA). Afterward, an ordinary one-way ANOVA test of data with equal variances and Brown–Forsythe combined with Welch’s ANOVA test of data with unequal variances were conducted to compare the values and to determine statistical differences between the study groups or the different time points. All multiple comparison tests were combined with false discovery rate (FDR) correction. Statistical differences were stated as significant if *p*-values were less than 0.05 and highly significant if *p*-values were less than 0.01, less than 0.001, or less than 0.0001. Finally, the data were graphed as means and standard deviations. Intraindividual differences were symbolized with asterisks (*) (from one to four asterisks according to the *p*-value). Similarly, interindividual differences were indicated with either hashtags (#) for differences between 2- and 16-week datasets, pluses (+) for differences between 2- and 8-week datasets, or with dots (•) for differences between 8- and 16-week datasets.

Additionally, correlation analyses were performed based on the multiple variables measured in the present study. Correlation matrix analyses (two-tailed Pearson’s correlation) were conducted to uncover whether a relationship existed between two parameters within the same compartment or between two compartments that lay above each other. Pearson’s correlation coefficient (r) indicated whether the parameters were either positively, negatively, or weakly correlated. In this paper, only positive correlations were considered. In this case, the *p*-value was also taken under consideration as a measurement of how significant the correlation was, which is indicated in the heatmaps. The confidence interval for these analyses was 95%.

## Figures and Tables

**Table 1:** Histomorphometrical measurement results of the tissue distribution. Values are the percentage of the TIA. MCBG: membrane compartment above bone graft-filled bone defects; MCSO: membrane compartment above sham operation; BG: bone graft-filled bone defect; and SO: sham operation bone defects.

	Tissue Fraction	MCBG	MCSO	BG	SO
2 weeks	Bone tissue (%)	1.6 ± 3.7	1.6 ± 2.1	22.8 ± 12.5	15.0 ± 15.3
	Biomaterial (%)	24.7 ± 12.7	28.6 ± 8.2	10.3 ± 7.5	-
8 weeks	Bone tissue (%)	35.5 ± 10.9	26.6 ± 9.4	33.4 ± 16.1	51.6 ± 25.7
	Biomaterial (%)	14.5 ± 13.1	9.9 ± 13.9	19.3 ± 9.2	-
16 weeks	Bone tissue (%)	33.4 ± 12.7	27.9 ± 17.3	34.0 ± 23.4	48.8 ± 28.6
	Biomaterial (%)	4.2 ± 2.1	9.1 ± 11.6	15.2 ± 10.4	-

**Table 2:** Macrophage subtype numbers at 2, 8, and 16 weeks post implantationem. MCBG: membrane compartment above bone graft-filled defects; MCSO: membrane compartment above sham operation; BG: bone graft-filled defects; and SO: sham operation bone defect.

	MCBG	MCSO	BG	SO
CD163 (cells/mm^2^)				
2 weeks	1201 ± 571.9	1006 ± 562.6	308.7 ± 146.9	371.8 ± 244.3
8 weeks	1100 ± 474.9	823.5 ± 413.8	365.3 ± 173.8	346.5 ± 189.8
16 weeks	691.5 ± 320.8	987.8 ± 558.2	241.1 ± 122.0	297.8 ± 110.1
CD11c (cells/mm^2^)				
2 weeks	503.7 ± 126.8	422.6 ± 170.4	8.8 ± 10.0	7.6 ± 4.6
8 weeks	290.5 ± 190.6	212.4 ± 115.9	16.6 ± 11.8	20.0 ± 11.2
16 weeks	104.2 ± 47.0	129.5 ± 39.4	14.6 ± 11.5	11.51 ± 5.7

**Table 3:** Vascularization values (vessel density and vascularization percentage) at 2, 8, and 16 weeks post implantationem. MCBG: membrane compartment above bone graft-filled defects; MCSO: membrane compartment above sham operation; BG: bone graft-filled defect; and SO: sham operation bone defect.

	MCBG	MCSO	BG	SO
Vessel Density (vessels/mm^2^)				
2 weeks	291.2 ± 49.4	253.9 ± 42.9	323.8 ± 73.5	315.0 ± 48.8
8 weeks	184.8 ± 105.7	161.2 ± 80.7	249.7 ± 96.5	246.1 ± 55.8
16 weeks	202.8 ± 82.2	281.8 ± 83.4	183.1 ± 40.8	259.5 ± 70.7
Vascularization Percentage (%)				
2 weeks	5.2 ± 1.9	4.1 ± 1.0	7.2 ± 2.5	5.6 ± 1.6
8 weeks	2.2 ± 1.7	1.4 ± 0.8	4.6 ± 2.4	2.7 ± 1.1
16 weeks	1.9 ± 0.9	2.9 ± 1.4	2.6 ± 1.4	3.7 ± 1.5

## Data Availability

All data are included in the manuscript.

## Supplementary Materials

The following supporting information can be downloaded at: https://www.mdpi.com/article/10.3390/ijms24076833/s1.
