# Supplementary material for: Analyses of the Cellular Interactions between the Ossification of Collagen-Based Barrier Membranes and the Underlying Bone Defects
Source: Int J Mol Sci. 2023 Apr 6;24(7):6833. doi: 10.3390/ijms24076833 (PMC10095555; doi:10.3390/ijms24076833)
Supplement: Supplementary file 1 [file ijms-24-06833-s001.zip › ijms-2292087-supplementary.pdf]

Analyses of the Cellular Interactions between the Ossification of Collagen-Based Barrier Membranes and the Underlying Bone Defects

Supplementary Figures

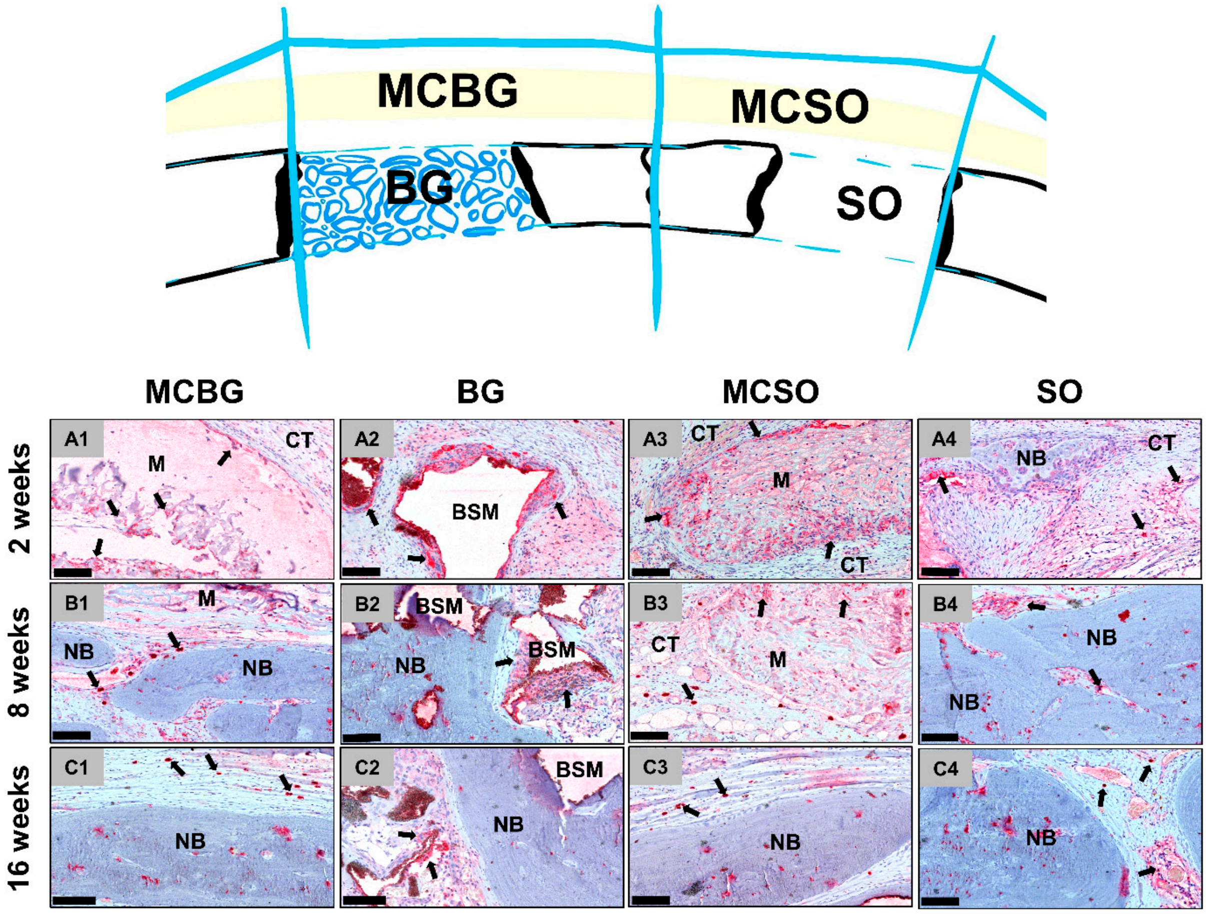

Supplementary Figure S1. Histological images of the pro-inflammatory immune response in the four compartments at (**A1**-**A4**) 2, (**B1**-**B4**) 8, and (**C1**-**C4**) 16 weeks *post implantationem*. Left row: Tissue responses within the membrane compartment above the implant defect (MCBG). Left center row: Tissue responses within the BSM-filled bone defects (BG). Right center row: Tissue responses within the membrane compartment above the sham operation defects (MCSO). Right row: Tissue responses within the sham bone defects (SO). M = membrane, BSM = bone substitute material, NB = new bone, CT = connective tissue, and black arrows = positive cells. (CD11c-immunostaining, 200× magnification, scale bars = 100 µm).

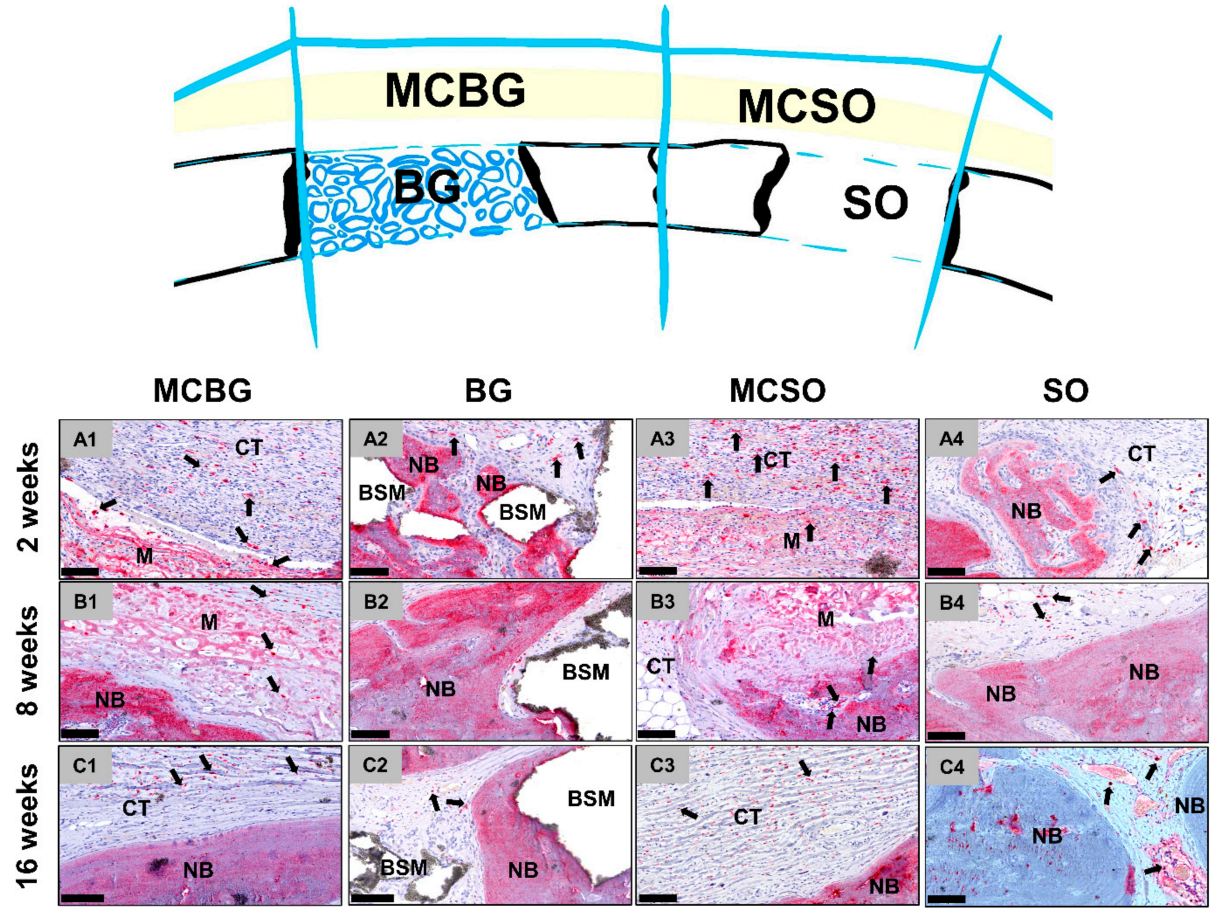

Supplementary Figure S2. Histological images of the anti-inflammatory immune response in the four compartments at (**A1**-**A4**) 2, (**B1**-**B4**) 8, and (**C1**-**C4**) 16 weeks *post implantationem*. Left row: Tissue responses within the membrane compartment above the implant defect (MCBG). Left center row: Tissue responses within the BSM-filled bone defects (BG). Right center row: Tissue responses within the membrane compartment above the sham operation defects (MCSO). Right row: Tissue responses within the sham bone defects (SO). M = membrane, BSM = bone substitute material, NB = new bone, CT = connective tissue, and black arrows = positive cells. (CD163-immunostaining, 200× magnification, scale bars = 100 µm).

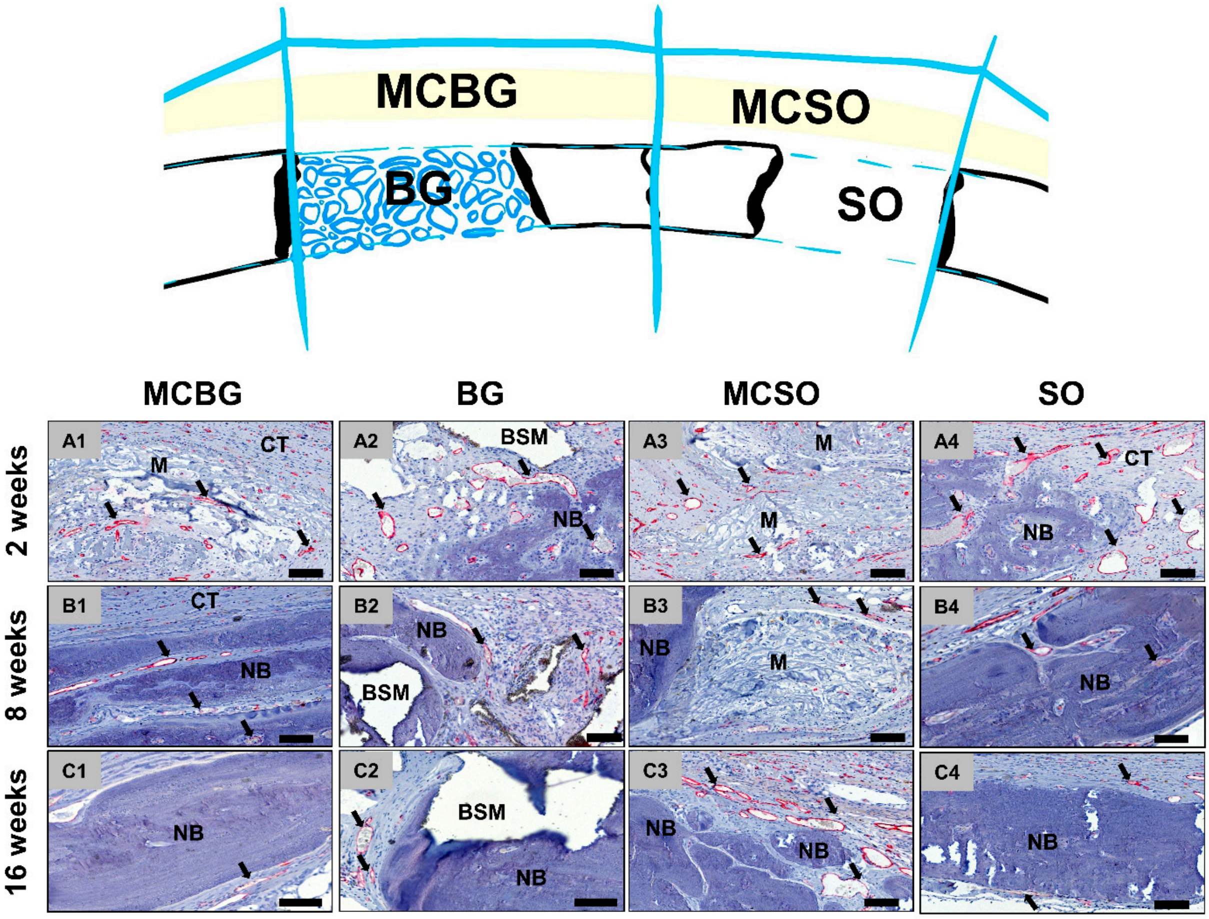

Supplementary Figure S3. Histological images of the vascularization patterns in the four compartments at (**A1**-**A4**) 2, (**B1**-**B4**) 8, and (**C1**-**C4**) 16 weeks *post implantationem*. Left row: Vessels within the membrane compartment above the implant defect (MCBG). Left center row: Vessels within the BSM-filled bone defects (BG). Right center row: Vessels within the membrane compartment above the sham operation defects (MCSO). Right row: Vessels within the sham bone defects (SO). M = membrane, BSM = bone substitute material, NB = new bone, CT = connective tissue, and black arrows = positive cells. (CD31-immunostaining, 200× magnification, scale bars = 100 µm).

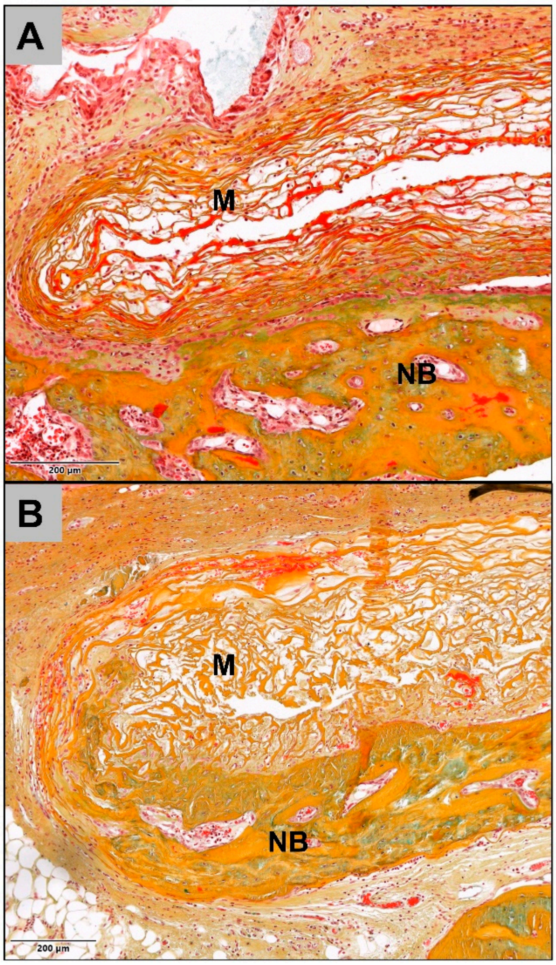

Supplementary Figure S4. Exemplary histological images showing the transition of collagen to bone tissue at **(A)** 2 and **(B)** 8 weeks. M = membrane, NB = new bone. (Movat's Pentachrome-staining, 130× magnification).
